# Association of RDM1 with osteosarcoma progression via cell cycle and MEK/ERK signalling pathway regulation

**DOI:** 10.1111/jcmm.16735

**Published:** 2021-07-15

**Authors:** Jun Sheng, Kun Liu, Dawei Sun, Piming Nie, Zhiping Mu, Hui Chen, Zhengfeng Zhang

**Affiliations:** ^1^ Department of Orthopedics Xinqiao Hospital Army Medical University Chongqing China; ^2^ Department of Orthopedic Surgery PLA Rocket Force Characteristic Medical Center Beijing China; ^3^ College of Life Sciences Zhejiang University Hangzhou China

**Keywords:** cell cycle, MEK/ERK signalling pathway, Osteosarcoma, RDM1

## Abstract

RAD52 motif‐containing 1 (RDM1), a key regulator of DNA double‐strand break repair and recombination, has been reported to play an important role in the development of various human cancers, such as papillary thyroid carcinoma, neuroblastoma and lung cancer. However, the effect of RDM1 on osteosarcoma (OS) progression remains unclear. Here, this study mainly explored the connection between RDM1 and OS progression, as well as the underlying mechanism. It was found that RDM1 was highly expressed in OS cells compared with human osteoblast cells. Knockdown of RDM1 caused OS cell proliferation inhibition, cell apoptosis promotion and cell cycle arrest at G1 stage, whereas RDM1 overexpression resulted in the opposite phenotypes. Furthermore, RDM1 silencing leads to a significant decrease in tumour growth in xenograft mouse model. RDM1 also increased the protein levels of MEK 1/2 and ERK 1/2. All these findings suggest that RDM1 plays an oncogenic role in OS via stimulating cell cycle transition from G1 to S stage, and regulating MEK/ERK signalling pathway, providing a promising therapeutic factor for the treatment of OS.

## 1 INTRODUCTION

RAD52 motif‐containing 1 (RDM1), a key regulator of DNA double‐strand break repair and recombination, is located at 17q11.2 and belongs to gene‐binding motif‐containing family.^1^ RDM1 contains RD motif, which is a stretch of 21 amino acid motif that shares with RAD52, and plays a vital role in DNA damage repair pathway. RDM1 binds to ssDNA and double‐stranded DNA. Furthermore, RDM1 recognizes the double helix distortions and involves in DNA double‐strand break repair and recombination events, RNA processing and protein translation.^2^ In recent studies, RDM1 is found to be abnormally expressed in various human cancers, playing a key role in cancer progression. In clinical hepatocellular carcinoma (HCC) samples, low expression of RDM1 is associated with larger tumour size, poor tumour differentiation and unfavourable survival.^3^ In papillary thyroid carcinoma, RDM1 is up‐regulated, and RDM1 knockdown caused cell proliferation inhibition, cell apoptosis induction and cell cycle arrest in the G2/M phase.^1^ In neuroblastoma, stable knockdown of RDM1 suppresses tumour growth in vivo, and RDM1 promotes cell proliferation through regulating RAS‐Raf‐Mek‐ERK pathway.^4^ In lung cancer, RDM1 is highly expressed in cancer cell lines and tissues, and its overexpression is reported to be correlated with histological differentiation, tumour size, lymph node metastasis and tumour‐node‐metastasis (TNM) stage, suggesting RDM1 potentially repairs DNA double‐strand breaks arising through DNA replication, thereby preventing G2/M cell cycle arrest.^5^ Additionally, the lung adenocarcinoma patients with higher mRNA expression of RDM1 show the worse clinical outcome.^6^ In both ovarian carcinoma and breast cancer, RDM1 also plays an oncogenic role,^7^ whereas in hepatocellular carcinoma, RDM1 expression is decreased, and RDM1 silencing enhances cancer procession via p53 and Ras/Raf/ERK signalling pathway.^3^ Importantly, RDM1 inhibition is reported to increase the sensitivity of cells to cisplatin,^8^ suggesting it may be used as an adjuvant therapy for cancer treatment. However, the influence of RDM1 on osteosarcoma (OS) remains unclear.

OS, the most common malignant tumour of bone in children and adolescents, has a peak occurring during the second and third decades of life.^9^ OS is derived from primitive mesenchymal cells and originates from bone and only rarely from soft tissue.^10^ Patients with OS frequently develop tumour metastasis in the absence of treatment, and 90% of them have died from pulmonary metastases before the introduction of polychemotherapy.^10^ The 5‐year survival rate has been improved to 60%‐70% for patients with localized osteosarcoma and 20%~30% for patients with metastases with the treatment of surgery and chemotherapy.^11^ However, this rate has not been increased over the last 30 years, and 80% of patients will still develop local relapse or metastatic disease even after surgical treatment. Although a serious of oncogene and suppressor genes have been identified, OS still lacks specific tumour markers. Furthermore, in most patients, the aetiology of OS remains obscure. Therefore, exploring the underlying molecular mechanisms of OS progression is of great importance.

In this study, we mainly investigated the roles of RDM1 in OS progression and the molecular mechanism involved. Abnormally, high expression of RDM1 was observed in OS tissues and cancer cells. Importantly, RDM1 knockdown lead to cell proliferation inhibition, cell apoptosis induction and cell cycle arrest at G1 stage. Also, RDM1 silencing decreased tumour growth in vivo, whereas RDM1 overexpression resulted in the opposite phenotypes. RDM1‐induced activation of MEK/ERK signalling pathway may be one of the underlying mechanisms for these changes.

## 2 METHODS AND MATERIALS

### 2.1 Clinical samples

A total number of 11 OS tissues and adjacent tissues were obtained from Xinqiao Hospital. Informed consent was signed by all the patients. Tissues were frozen in liquid nitrogen and stored at −80°C before experiments. All samples were collected in accordance with ethical guidelines, and written informed consent was received. All patients were approached based on approved ethical guidelines, and patients who agreed to participate in this study were required to sign consent forms before being included in the study. All experimental protocols and methods were approved by Medical ethical committee of Xinqiao Hospital (Ethics number: 20200905).

### 2.2 Cell lines and culture

Three OS cell lines (143B, SaoS2 and MG63) and one human osteoblast cell line (hFOB1.19) were purchased from American Type Culture Collection (ATCC) and were cultured in DMEM (Dulbecco's modified Eagle's medium; Gibco; Invitrogen) supplemented by 10% FBS (foetal bovine serum; Gibco, USA), and maintained in a humidified atmosphere incubator at 37°C in a 5% CO_2_ atmosphere.

### 2.3 Cell transfection

Cells were seeded into six‐well plates and incubated overnight at 37°C in a 5% CO2 atmosphere. After reaching 40‐50% confluence, cells were transfected with normal control (NC), target small interfering RNA (siRNA) or overexpression plasmid using the Lipofectamine 2000 (Thermo Fisher Scientific), according to the manufacturer's instructions.

### 2.4 RNA extraction and quantitative real‐time PCR (qRT‐PCR)

Total RNA from OS tissues or cells was extracted by using TRIzol reagent (Invitrogen; Thermo Fisher Scientific) following the manufacturer's recommendation. After RNA concentration detection, cDNA was synthesized using the PrimeScript RT reagent kit (Takara Bio Inc), and qRT‐PCR was performed with SYBR Premix Ex Taq (Takara Bio Inc). *18S* was used as an internal standard.

### 2.5 Western blotting assay

Total proteins were isolated from cells by using lysis buffer. Protein concentration and quantity were measured using a BCA kit. After that, we use 10% sodium dodecyl sulphate (SDS)‐polyacrylamide gels to separate proteins and then transferred these proteins onto PVDF membranes. After blocking in fat‐free milk, membranes were incubated with primary antibodies specific for RDM1, GAPDH, caspase‐3, cleaved‐caspase‐3, MEK 1/2, ERK 1/2, p53, p21 or PCNA overnight. Then, they were washed with TBST and incubated with secondary antibodies. In the end, protein signals were evaluated using an enhanced chemiluminescence detection kit (EMD Millipore).

### 2.6 Colony formation assay

After transfection, cells were seeded in 6‐well culture plates for 2 weeks. After that, 1% crystal violet was used to stain cells for visualization. Colony images were taken and counted after washing and air drying.

### 2.7 Caspase 3/7 activity analysis

Caspase 3/7 activity was used to analyse cell apoptosis. After transfection, cells were seeded in 96‐well plate. Then, cells were collected and lysed by lysis buffer. After centrifugation, supernatant fluid was obtained. And then, it was mixed with reaction buffer and caspase 3/7 substrate. In the end, 405 nm absorbance was measured by SpectraMax M3 microplate reader (Molecular Devices).

### 2.8 Cell cycle analysis

Cells were collected and stained with propidium iodide (PI) and ribonuclease based on the manufacture's recommendation. DNA PI‐associated fluorescence in cells was determined using flow cytometer.

### 2.9 Animal experiments

Four‐week‐old female Balb/c nude mice were obtained. After cell transfection, U2OS cells with or without RDM1 knockdown were injected into the flank region of nude mice subcutaneously at 2 × 10^6^ cells in 100 μL per spot. Tumour volume was measured every 3 days by using a digital calliper. After 24 days, mice were killed, and tumour weight was measured. All experimental protocols and methods were approved by Medical ethical committee of Xinqiao Hospital (Ethics number: 20 200 905).

### 2.10 Statistical analysis

Statistically significant differences between groups were calculated using two‐tailed paired Student's t test or one‐way ANOVA. All values were expressed as the mean ± standard error of the mean (SEM) from three independent experiments. Statistics analysis was performed with GraphPad Prism software (version 8.0; GraphPad Prism Software, Inc). *P* < .05 was considered as statistically significant.

## 3 RESULTS

### 3.1 RDM1 was overexpressed in OS samples and cells

**FIGURE 1:**
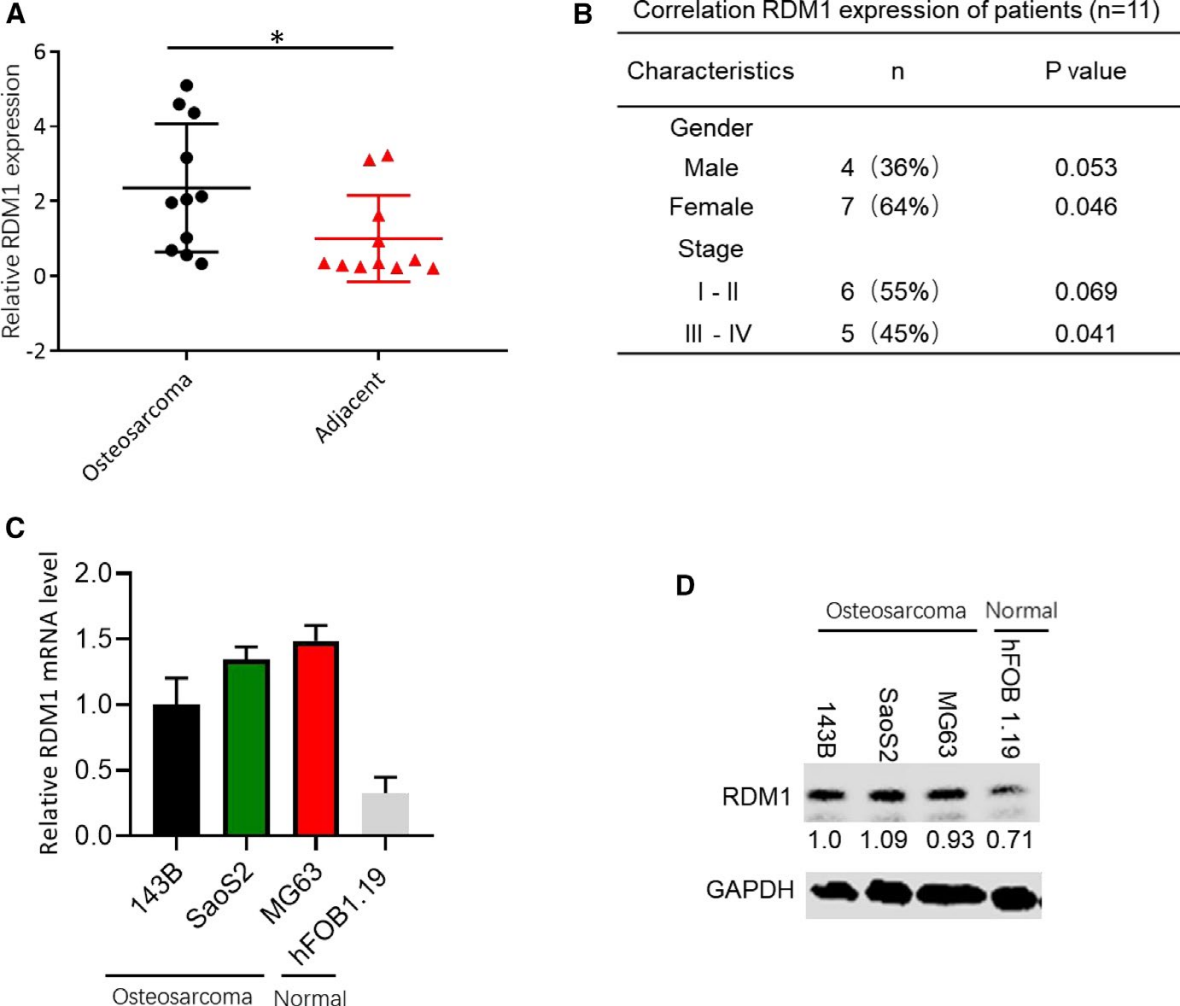
RDM1 was highly expressed in OS samples and cells. A, The expression levels of RDM1 in OS tissues and adjacent tissues. B, Correlation RDM1 expression of OS patients. C, The mRNA levels of RDM1 in OS cell lines (143B, SaoS2 and MG63) and normal human osteoblast cell (hFOB1.19). D, The protein levels of RDM1 in OS cell lines (143B, SaoS2 and MG63) and normal human osteoblast cell (hFOB1.19). OS, osteosarcoma; GAPDH was used as the control. Data are presented as the mean ± SEM of three independent experiments. **P < .01, ***P < .001

To investigate the expression of RDM1 in OS, 11 OS tissues and 11 adjacent tissues were obtained. The results showed that RDM1 was highly expressed in OS tissues compared with adjacent tissues (Figure 1A). In addition, the protein and mRNA levels of RDM1 in OS cell lines (143B, SaoS2 and MG63) were markedly increased compared with hFOB1.19, a human osteoblast cell (Figure 1B,C), indicating that RDM1 is associated with OS tumorigenesis.

### 3.2 RDM1 promoted OS cell proliferation

**FIGURE 2:**
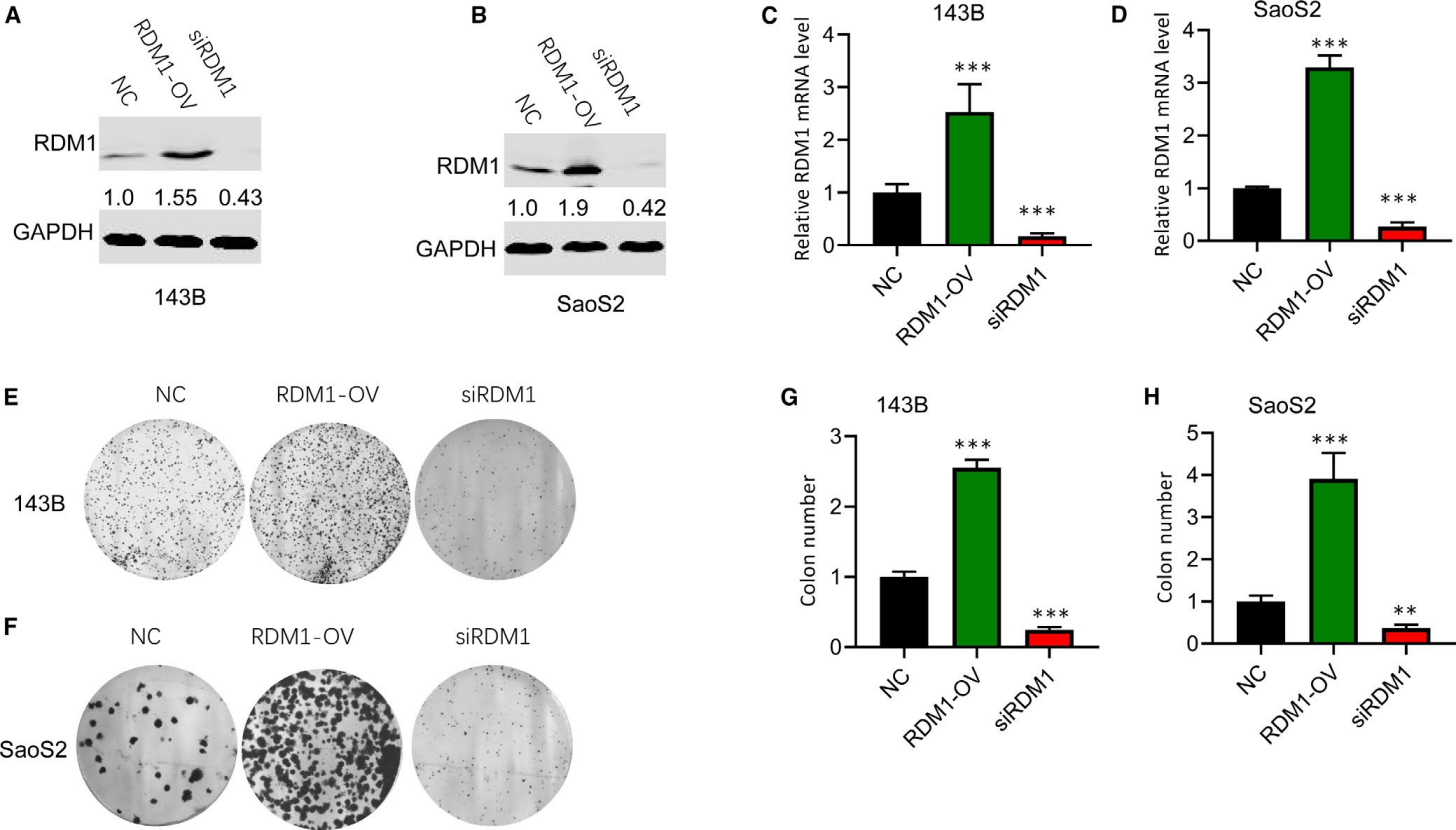
RDM1 accelerated cell proliferation in 143B and SaoS2 cells. A‐B, Down‐regulation and overexpression of RDM1 in (A) 143B and (B) SaoS2 cells were verified by Western blotting assay after transfection with NC, siRDM1 or RDMI‐OV plasmid for more than 36 h. C‐D, the mRNA levels of RDM1 in (C)143B and (D) SaoS2 cells were validated by qRT‐PCR after transfection with NC, siRDM1 or RDMI‐OV plasmid for more than 36 h. E‐F, The cell proliferation was detected by colony formation assay in (E) 143B and (F) SaoS2 cells. G‐H, The statistical results of E (G) and F (H). NC, normal control; siRDM1, small interfering RDM1; RDMI‐OV, RDMI overexpression; GAPDH was used as the Western blotting control. Data are presented as the mean ± SEM of three independent experiments. **P < .01, ***P < .001

To explore the effect of RDM1 on the proliferation of OS cells, we transfected normal control (NC), siRDM1 or pcDNA3.1‐RDM1 into 143B and SaoS2 cells, and found RDM1 protein and mRNA levels were successfully silenced or up‐regulated in both cell lines (Figure 2A,B). The colony formation assay showed that up‐regulating RDM1 caused the increase in OS cell growth, and inhibiting RDM1 suppressed cell growth compared with normal control group (Figure 2E‐H). These findings clarified that RDM1 could regulate OS cell proliferation.

### 3.3 RDM1 induced the activation of MEK/ERK signalling pathway

**FIGURE 3:**
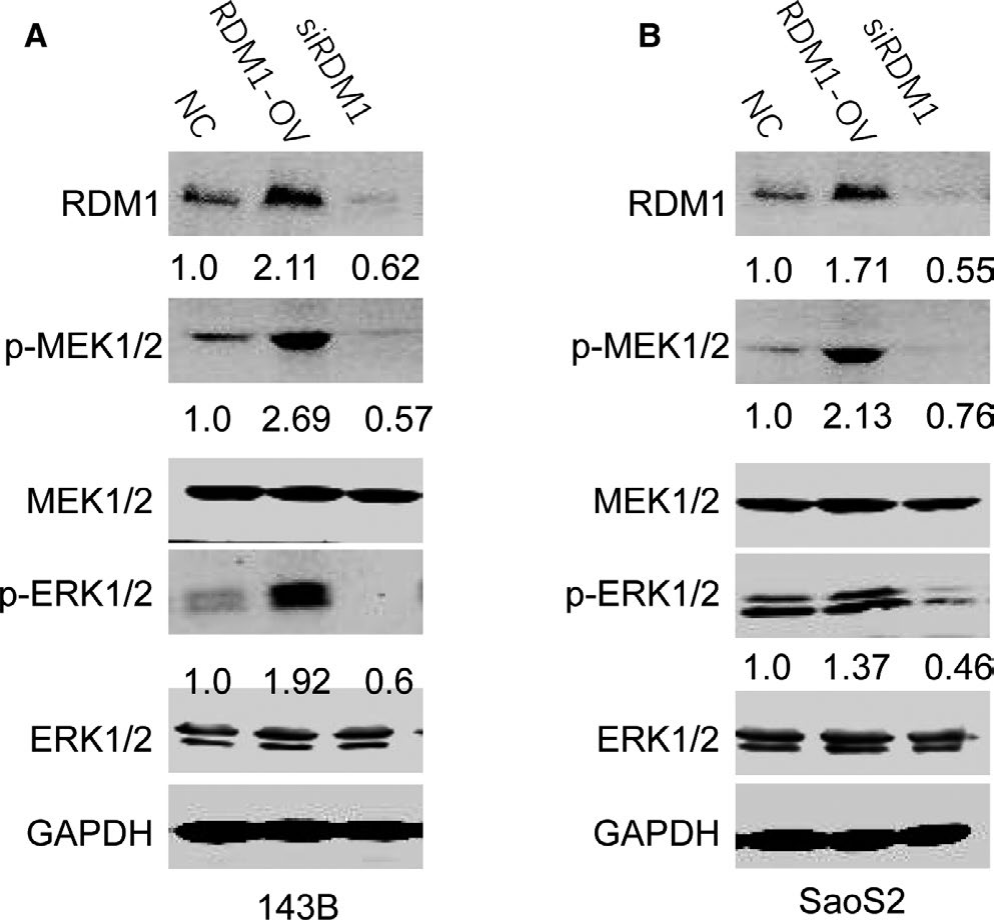
RDM1 induced MEK/ERK signalling pathway activation. A‐B, The protein levels of p‐MEK1/2, MEK1/2, p‐ERK1/2, ERK1/2 and RDM1 were verified by Western blotting assay in (A) 143B and (B) SaoS2 after transfection with NC, siRDM1 or RDMI‐OV plasmid for more than 36 h. NC, normal control; siRDM1, small interfering RDM1; RDMI‐OV, RDMI overexpression; GAPDH was used as the Western blotting control. Data are presented as the mean ± SEM of three independent experiments. **P < .01, ***P < .001

It is reported that MEK/ERK signalling pathway promotes cell proliferation.^12^ To investigate whether RDM1 promoted OS progression by regulating MEK/ERK signalling pathway, we detected the expression of p‐MEK 1/2 and p‐ERK 1/2 in cell lines with RDM1 knockdown or overexpression and found that overexpression of RDM1 increased the expression levels of p‐MEK 1/2 and p‐ERK 1/2 both in 143B and SaoS2 cell lines, indicating RDM1 induced MEK/ERK signalling pathway activation. Meanwhile, silencing RDM1 caused the reduction in p‐MEK 1/2 and p‐ERK 1/2 protein levels compared with normal control cells (Figure 3A,B). These findings validated that RDM1 regulated the activation of MEK/ERK signalling pathway in OS cells.

### 3.4 RDM1 inhibited OS cell apoptosis

**FIGURE 4:**
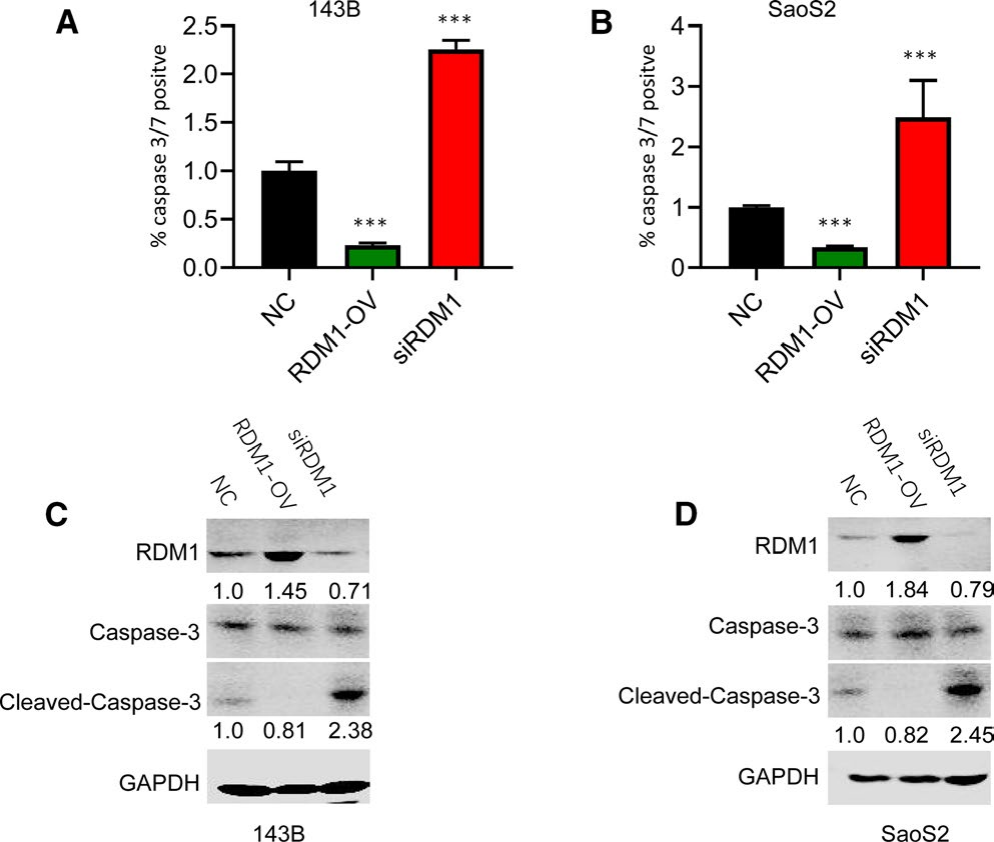
RDM1 inhibited cell apoptosis in 143B and SaoS2 cells. A‐B, The cell apoptosis was detected by caspase 3/7 activity assay in (A) 143B and (B) SaoS2 after transfection with siRDM1 or RDMI‐OV plasmid for more than 36 h. C‐D, The protein levels of caspase 3, cleaved caspase 3 and RDMI were verified by Western blotting assay in (A) 143B and (B) SaoS2 after transfection with NC, siRDM1 or RDMI‐OV plasmid for more than 36 h. NC, normal control; siRDM1, small interfering RDM1; RDMI‐OV, RDMI overexpression; GAPDH was used as the control. Data are presented as the mean ± SEM of three independent experiments. **P < .01, ***P < .001

Caspase 3/7 is a valuable biomarker for cell apoptosis.^13^ Therefore, by testing positivity of caspase 3/7, we can evaluate the degree of apoptosis. The results showed that silencing RDM1 significantly increased caspase 3/7 activity both in 143B and SaoS2 cells, while RDM1 overexpression inhibited its activity (Figure 4A,B). Furthermore, the protein levels of caspase 3 and cleaved caspase 3 were also examined. Western blotting assay showed that caspase 3 protein was mildly up‐regulated and cleaved caspase 3 was down‐regulated in cells with RDM1 overexpression. In addition, the result was totally reverse for cells with RDM1 knockdown (Figure 4C,D). All these findings suggested that overexpression of RDM1 inhibited cell apoptosis, which may be the reason for its promotion effect on cell proliferation in OS cell lines.

### 3.5 RDM1 promoted OS cell cycle transition from G1 to S stage

**FIGURE 5:**
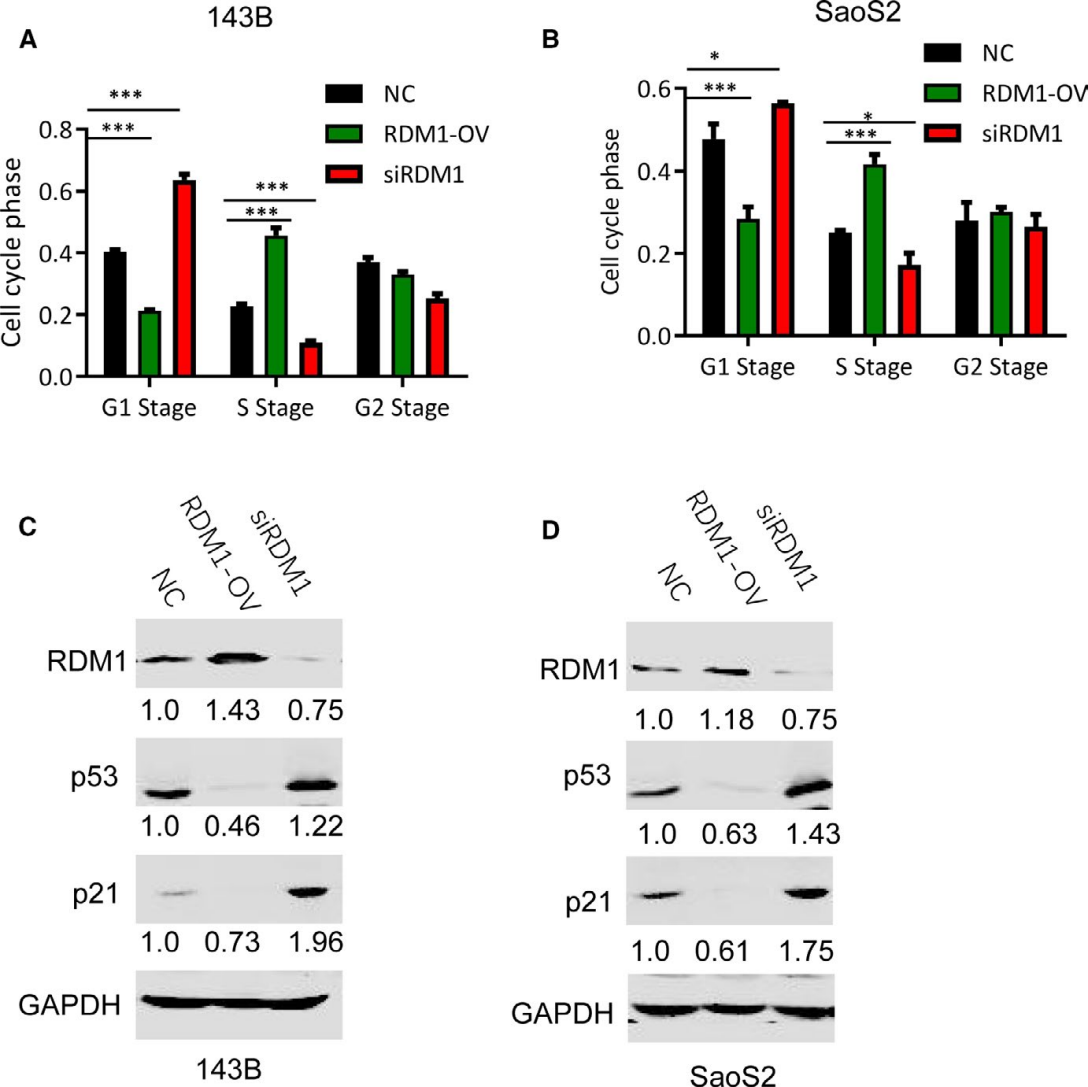
RDM1 promoted cell cycle transition from G1 to S stage. A‐B, The cell cycle analysis of (A) 143B and (B) SaoS2 after transfection with NC, siRDM1 or RDMI‐OV plasmid for more than 36 h. C‐D, The protein levels of p53, p21 and RDM1 were verified by Western blotting assay in (C) 143B and (D) SaoS2 cells after transfection with NC, siRDM1 or RDMI‐OV plasmid for more than 36 h. NC, normal control; siRDM1, small interfering RDM1; RDMI‐OV, RDMI overexpression; GAPDH was used as the control. Data are presented as the mean ± SEM of three independent experiments. **P < .01, ***P < .001

Cell cycle analysis was detected by flow cytometry. In this study, overexpression of RDM1 induced cell cycle transition from G1 to S stage, while RDM1 knockdown caused cell cycle arrest at G1 stage. However, there was no significant difference at G2 Stage in both 143B and SaoS2 cells (Figure 5A,B).

p53 and p21 are well‐known tumour suppressors and contribute to G1 stage arrest.^14^ Here, this study further detected the role of RDM1 on the protein levels of p53 and p21. Overexpression of RDM1 significantly decreased their levels in both 143B and SaoS2 cell lines, and knockdown of RDM1 increased p53 and p21 protein levels (Figure 5C,D). These findings validated the roles of RDM1 in cell cycle.

### 3.6 RDM1 knockdown decreased osteosarcoma growth in xenograft mouse model

**FIGURE 6:**
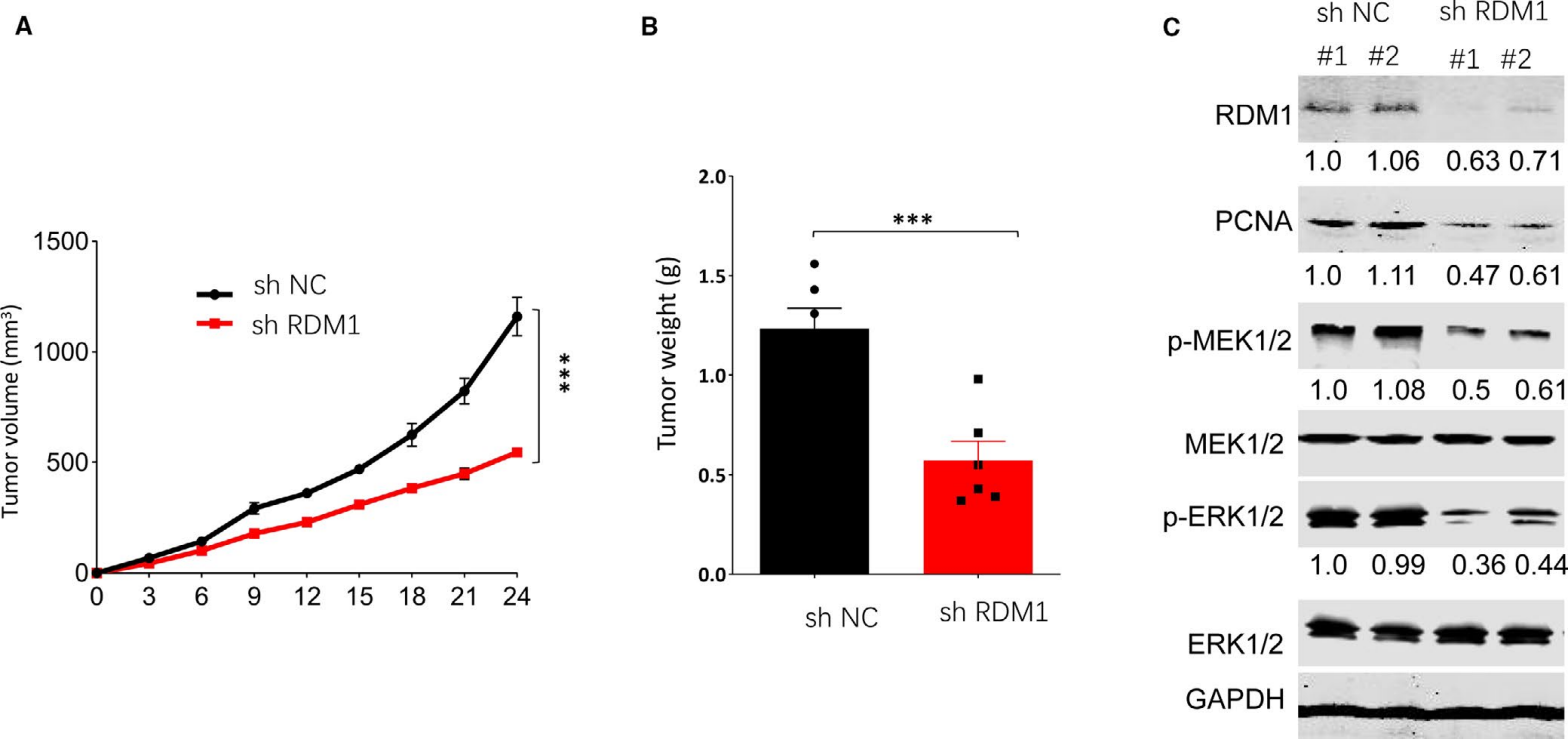
RDM1 silencing inhibited tumour growth in vivo. A, Tumour volume statistics in mice after being injected with shRDM1 U2OS cells or shNC U2OS cells during 24 days. B, Tumour weight statistics in mice after being injected with shRDM1 U2OS cells or shNC U2OS cells for 24 days. C. The protein levels of p‐MEK1/2, MEK1/2, p‐ERK1/2, ERK1/2, RDM1 and PCNA were verified by Western blotting assay in tumours. shNC, short hairpin Normal control; shRDM1, short hairpin RDM1; GAPDH was used as the control. Data are presented as the mean ± SEM of three independent experiments. **P < .01, ***P < .001

To further investigate the effect of RDM1 on OS growth in vivo, U2OS cells transfected with shNC or shRDM1 were injected into the flank region of nude mice subcutaneously. The result in vivo was consistent with that in vitro. Silencing RDM1 significantly decreased tumour volume and weight, and inhibited tumour growth (Figure 6A,B).

PCNA is a marker for cell proliferation and cancer virulence, and its expression is always up‐regulated in cancer cells.^15^ This study then tested PCNA level in OS and found that inhibition of RDM1 suppressed PCNA expression compared with normal control group (Figure 6C), indicating that knockdown of RDM1 inhibited tumour group, which is consistent with the results of tumour volume and tumour weight. Furthermore, we also measured the protein levels of p‐MEK 1/2 and p‐ERK 1/2, and found that inhibition of RDM1 suppressed p‐MEK 1/2 and p‐ERK 1/2 expression compared with normal control group (Figure 6C), which was consistent with that in vitro.

## 4 DISCUSSION

RAD52 motif‐containing 1 (RDM1), belonging to gene‐binding motif‐containing family, has been reported to have similarities with RAD52 which is an important regulator in DNA repair pathways.^6^ Excessive activation of RDM1 has been found to be involved in many human cancers, such as papillary thyroid carcinoma, neuroblastoma and lung cancer.^16^ However, few studies focus on the roles of RDM1 in OS development. Therefore, by using cell experiments in vitro and mice trial in vivo, we explored the effects of RDM1 on OS development and the underlying mechanism. The results showed that RDM1 plays an oncogenic role in human OS.

In detail, this study first found RDM1 was highly expressed in OS tissues and cell lines, which was consistent with previous studies.^16^ Subsequently, RDM1 silencing not only suppressed cell proliferation in vitro but also inhibited tumour growth in vivo. In addition, suppression of RDM1 promoted cell apoptosis in 143B and SaoS2 cell lines by analysing caspase 3/7 activity as well as the protein levels of caspase 3 and cleaved caspase 3. Li W et al,^1^ Xie G et al^4^ and Xu G et al^5^ also found that RDM1 knockdown caused cell proliferation inhibition, and cell apoptosis induction in papillary thyroid carcinoma, neuroblastoma, and lung cancer, respectively.

In this study, RDM1 promoted cell cycle transition from G1 to S stage using flow cytometry. Furthermore, p53 and p21 protein expression levels were detected. *P53* gene, which is located on the short arm of chromosome 17p13, encodes a tumour suppressor protein containing transcriptional activation, DNA binding and oligomerization domains. p53 is a tetrameric transcription factor regulating a variety of gene expression involved in cell apoptosis and cell cycle arrest. One of the key target genes regulated by p53 in cell cycle arrest is *P21*, and p53 can directly bind to *P21* promoter. *P21*, a cyclin‐dependent kinase inhibitor, is located on chromosome 6p21.2.^17^ p53 and p21 are important negative regulators for cell cycle progression by arresting G1 checkpoint.^18^ G1/S checkpoint takes charge of controlling S stage entry, which is lost in most human tumours. Cells, which are short of G1/S stage checkpoint, can start synthesizing DNA under non‐permissive conditions, leading to DNA damage.^19^ This study found that inhibition of RDM1 caused the reduction in p53 and p21 protein levels compared with normal control (NC) group, leading to G1 checkpoint arrest. That is, RDM1 silencing leads to cell cycle arrest at G1 stage. These findings suggest that RDM1 promoted OS cell proliferation and inhibited cell apoptosis via promoting cell cycle transition from G1 to S stage.

To explore the molecular mechanism underlying the oncogenic effects of RDM1 on OS, this study further examined the roles of RDM1 in MEK/ERK signalling pathway. ERK, an extra‐cellular signal‐regulated kinase, is a member of mitogen‐activated protein kinase family, which is activated by its upstream kinase, MEK1 and MEK2.^20^ ERK has been reported to act as the targets of 180 different molecules and is involved in many diverse cellular functions, such as cell growth, cell death, cell proliferation, cell apoptosis, cell transcription and adhesion. ERK can also play these roles in various cancers, such as lung, colorectal, breast and prostate cancer.^21^ More and more researches have proved that MEK‐ERK pathway contributes to the execution of cellular DNA damage response (DDR), a major pathway for tumour suppression. Furthermore, MEK‐ERK signalling pathway is activated during DDR, promoting the activation of DDR checkpoints to suppress cell division.^22^ In the present study, we found overexpression of RDM1 remarkably increased p‐MEK 1/2 and p‐ERK 1/2 protein levels in 143B and SaoS2 cell lines compared with normal control group. On the contrary, RDM1 silencing inhibited p‐MEK 1/2 and p‐ERK 1/2 protein expression. In neuroblastoma, RDM1 is also reported to increase the protein levels of p‐ERK and p‐MEK.^4^ These findings suggest that RDM1 may promote OS progression via activating MEK/ERK signalling pathway.

## 5 CONCLUSION

Herein, this study indicated that RDM1 caused an increase in cell proliferation in vitro and tumour growth in vivo, as well as the inhibition of cell apoptosis via inducing cell cycle transition from G1 to S stage, and activating MEK/ERK signalling pathway. These findings identify RDM1 as an oncogene in OS and provide a promising strategy to treat OS in the future.

## ACKNOWLEDGEMENTS

This work was supported by Foundation of Science Technology Department of Sichuan Province [2018FZ0065].

## CONFLICT OF INTEREST

All the authors declare that there is no conflict of interest.

## AUTHOR CONTRIBUTIONS

**Jun Sheng:** Conceptualization (equal); Investigation (equal). **Kun Liu:** Investigation (equal). **Dawei Sun:** Conceptualization (equal); Investigation (equal). **Piming Nie:** Investigation (equal). **Zhiping Mu:** Investigation (equal). **Hui Chen:** Data curation (equal); Project administration (equal); Writing‐review & editing (equal). **Zhengfeng Zhang:** Conceptualization (equal); Writing‐review & editing (equal).
